# Self-assembled peptide/polymer hybrid nanoplatform for cancer immunostimulating therapies

**DOI:** 10.1007/s13346-023-01410-y

**Published:** 2023-09-18

**Authors:** Saeedeh Khazaei, Ruben Varela-Calviño, Mazda Rad-Malekshahi, Federico Quattrini, Safura Jokar, Nima Rezaei, Saeed Balalaie, Ismaeil Haririan, Noemi Csaba, Marcos Garcia-Fuentes

**Affiliations:** 1 Department of Pharmaceutical Biomaterials and Medical Biomaterials Research Center, Faculty of Pharmacy, Tehran University of Medical Sciences, Tehran, Iran; 2 Department of Pharmacology, Pharmacy and Pharmaceutical Technology, CiMUS Research Center and Health Research Institute of Santiago de Compostela (IDIS), University of Santiago de Compostela, Santiago de Compostela, Spain; 3 Department of Biochemistry and Molecular Biology, School of Pharmacy, University of Santiago de Compostela, Santiago de Compostela, Spain; 4 Department of Nuclear Pharmacy, Faculty of Pharmacy, Tehran University of Medical Sciences, Tehran, Iran; 5 Department of Immunology, School of Medicine, Tehran University of Medical Sciences, Tehran, Iran; 6 Peptide Chemistry Research Center, K. N. Toosi University of Technology, Tehran, Iran

**Keywords:** Cancer vaccine, Self-assembling peptide, Nanovaccines, Hybrid nanoparticles, MAGE-A3

## Abstract

Integrating peptide epitopes in self-assembling materials is a successful strategy to obtain nanovaccines with high antigen density and improved efficacy. In this study, self-assembling peptides containing MAGE-A3/PADRE epitopes were designed to generate functional therapeutic nanovaccines. To achieve higher stability, peptide/polymer hybrid nanoparticles were formulated by controlled self-assembly of the engineered peptides. The nanoparticles showed good biocompatibility to both human red blood- and dendritic cells. Incubation of the nanoparticles with immature dendritic cells triggered immune effects that ultimately activated CD8 + cells. The antigen-specific and IgG antibody responses of healthy C57BL/6 mice vaccinated with the nanoparticles were analyzed. The in vivo results indicate a specific response to the nanovaccines, mainly mediated through a cellular pathway. This research indicates that the immunogenicity of peptide epitope vaccines can be effectively enhanced by developing self-assembled peptide-polymer hybrid nanostructures.

**Graphical Abstract:**

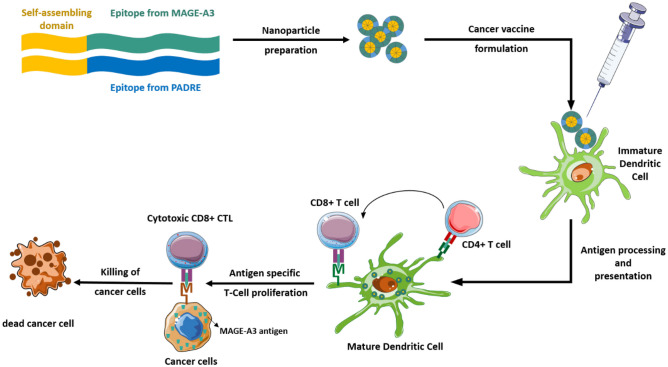

**Supplementary Information:**

The online version contains supplementary material available at 10.1007/s13346-023-01410-y.

## Introduction

Cancer is one of the most important causes of death worldwide accounting for 9.6 million casualties in 2018. The most established therapies for cancer are chemotherapy and radiotherapy, which have adverse side effects on normal cells and are ineffective to produce a durable protection, especially in the case of malignant tumors. Cancer immunotherapy has shown to be effective to fight against micro-metastases, and to produce a long-lasting immune response. However, there is still a huge demand to find new cancer immunotherapies with less side effects and reduced response variability across patients.

Controlling the development and progression of tumor cells, known as immune surveillance, is a physiologic function of the immune system [1]. Adaptive immune protection against tumor cells and their elimination is mediated by CD8 + cytotoxic T lymphocytes (CTLs). These cells are programmed to recognize and kill malignant cells that display peptide tumor epitopes within a class I MHC complex. Antigen Presenting Cells (APCs), particularly dendritic cells, ingest tumor cells and process their antigens to smaller peptide sequences. These peptides are bound to the class I MHC molecules peptide binding groove and exhibited on the APC surface for recognition by CD8 + T cells. CD4 + helper T (Th) cells role in anti-tumor immune responses is mediated by the secretion of several cytokines, such as tumor necrosis factor (TNF) and interferon-γ (IFN-γ), which activate macrophages and improve CD8 + T cell responses [2]. The pan-DR epitope (PADRE) is a general Th epitope able to bind to most of the MHC class II alleles. It has been extensively used in the vaccine development research to enhance the immune responses [3].

Over the last years, more than 70 proteins have been identified as tumor-associated antigens (TAAs). The whole protein structure of these antigens or a collection of their antigenic epitopes could be used in a therapeutic vaccine. The ideal TAAs for vaccination are those that are overexpressed in tumors compared to normal tissues [4, 5]. Cancer-testis antigens (CTA), including MAGE, GAGE, BAGE, and NYESO-1, are a group of TAAs shared among different tumor types, but absent in normal somatic cells except in testis and placenta. It is essential to point out that since MHC I is not expressed by testis and placenta cells, CTAs are not presented on their surface. MAGE-A3 is a CTA expressed in different cancer types, especially, melanoma and non-small cell lung cancer (NSCLC) [6, 7], and is a promising candidate TAA for vaccine development. Indeed, DERMA and MAGRIT are phase III clinical trials based on MAGE-A3 conducted on patients with melanoma skin cancer and NSCLC, respectively. In MAGRIT (NCT00480025) a recombinant MAGE-A3 together with the AS15 adjuvant, was administrated intramuscularly to 1515 NSCLC patients. This trial could not meet its primary endpoint of improving overall patient survival [8], indicating that new strategies for the delivery of TAAs are needed.

Proteins have complex supramolecular structures and might comprise different subunits. It is known that only specific sequences in a polypeptide chain are effective antigenic epitopes. These sequences can be identified and applied as antigenic epitopes instead of the whole protein. Feltkamp et al. were the first to prevent HPV tumor growth by immunizing mice with an E7 peptide vaccine [9]. However, several studies have demonstrated unspecific binding of short peptide vaccines to MHC molecules on the surface of any cell, which make them prone to induce T-cell tolerance and anergy [10, 11]. Synthetic long peptides are an alternative to short peptide vaccines, but they have shown limited induction of immune responses, especially due to degradation by blood proteases and endo-/ exopeptidases on the surface of DCs, which result in their rapid clearance from the injection site. Besides, long peptides usually show poor uptake by DCs [12, 13].

Improved delivery of peptide epitopes to DCs could be achieved by using nanoparticles that enhance their uptake and that provide a steric shield towards enzymatic degradation [14–19]. However, the problems associated with nanoparticulate systems, including low loading capacity and difficult fabrication, have limited their application in clinical trials and the pharmaceutical market. Self-assembling (SA) peptides are biodegradable carriers of considerable interest as biomaterials for drug delivery and immunotherapy [19]. These peptides associate in a structured way by intramolecular interactions when dispersed in an aqueous solvent. By connecting SA blocks to an epitope, particles with a high density of surface-exposed epitopes can be formed in an aqueous media [20]. In contrast to vaccines based on virus-like-particles, where the viral peptides could be immunogenic but toxic, the SA domain of these peptide vaccines is perfectly biocompatible, and the immune system interacts only with the incorporated epitopes [21, 22]. Some studies have shown that incorporating the peptide epitopes to a self-assembling fragment can effectively enhance the desired immune response by minimizing the possibility of T cell anergy and increasing cellular uptake. In 2016, a vaccine delivery system was designed by Hennink et. al., by introducing a SA peptide (Ac-AAVVLLLW-COOH) to the N-terminal of peptide epitopes from OVA and HPV-E7. Administration of a vaccine formulation containing these peptide nanoparticles and a CpG adjuvant to tumor-bearing mice could efficiently stimulate antigen-specific CD8 + T cells, which in turn reduced the tumor growth and improved the survival in a C57BL/6 mice [23].

In this study, a cancer vaccine was designed by connecting peptide epitopes MAGE-A3 and PADRE to the N-terminal of a self-assembling peptide. These peptides were used to generate nanovaccines by simple dispersion in a water/poloxamer solution. The resulting structures were characterized pharmaceutically and tested for their capacity to induce tumor antigen-specific immune responses in cell culture and in a murine model.

## Materials and methods

### Materials

SATC and SATH peptides were purchased from ChinaPeptides (Shanghai, China). The side-chain protected Fmoc-amino acids were obtained from Zhejiang Materials Industry Co. (Zhejiang, China). 2-chlorotrityl chloride (2-CTC) resin, 2-(1H-benzotriazol-1-yl)-1,1,3,3-tetramethyluronium tetrafluoroborate (TBTU) and N, N-diisopropylethylamine (DIPEA) were purchased from GL Biochem. (Shanghai, China). Pluronic^®^ F127 (F127) was obtained from BASF (Ludwigshafen, Germany). Potassium chloride (KCl), D-(+)-Trehalose dehydrate, Nile Red, *Escherichia coli* lipopolysaccharide (LPS)*,* complete and incomplete Freund’s adjuvant, Ficoll (Histopaque^®^-1083) and p-nitrophenol phosphate substrate solution (N2770-5SET) were purchased from Sigma-Aldrich (Missouri, USA). Ficoll-Paque™ PLUS (density 1.077 g/mL) was purchased from GE Healthcare Bio- Science AB (Illinois, USA). PBS and RPMI-1640 were obtained from GIBCO^®^ (Thermo Fisher Scientific, Massachusetts, USA). Granulocyte–macrophage colony-stimulating factor (GM-CSF), interleukin 4 (IL-4), allophycocyanin (APC)-conjugated anti-human CD83 (CD83- APC), phycoerythrin (PE)-conjugated anti-human CD80 (CD80-PE), and fluorescein (FITC)-conjugated anti-human CD1a (CD1a-FITC), were purchased from Miltenyi Biotec (Bergish Gladbach, Germany). Anti CD4-APC, anti CD25-PE, anti CD8a-APC, and anti CD28-PE antibodies were obtained from Tonbo Biosciences (California, USA). Goat Anti-Mouse IgG H&L (alkaline phosphatase, ab97020) were obtained from Abcam (Massachusetts, USA). Fetal bovine serum (FBS), PSG (100 U/mL penicillin, 0.1 mg/mL streptomycin, and 2 mM L-glutamine) were purchased from Invitrogen (CA, USA). MTS CellTiter 96^®^ AQueous Non-Radioactive Cell Proliferation Assay kit was provided by Promega (Madison, USA). Interferon gamma (IFN-γ) was purchased from Peprotech (London, UK). ELISPOT kit was purchased from U-CyTech (Biosciences, Utrecht, The Netherlands). All other chemicals were of reagent grade or higher purity.

### Methods

#### Vaccine design

Self-assembly sequence was added to the N-terminal of the cytotoxic T cell (TC) and Helper T cell (TH) epitopes using AAY and KFERQ linkers, respectively. The N-acetylated peptides which were used in this study are listed in Table 1. The selected peptide epitopes were tested for their characteristics in open web platforms. The binding affinity and processing of SATC and SATH peptides for the MHCI/MHCII pathways were calculated with the Immune Epitope Database (IEDB). The allergenicity of the designed vaccine was evaluated in AllerTOP v2.0 (http://www.ddg-pharmfac.net/AllerTOP/) server [24]. The toxic/non-toxic nature of peptides was predicted using the ToxinPred module (http://crdd.osdd.net/raghava/toxinpred/multi_submit.php) [25]. The prediction of the physicochemical characteristics of the vaccine was done using the ProtParam tool of the ExPASy database server (http://web.expasy.org/protparam/) [26].

**Table 1:** List of the peptide sequences used in this study

**Name**	**Sequence**	**Molecular weight** **(g/mol)**	**Net weight** **(%)**	**Function**
**TC**	Ac-KVAELVHFL	1055	83	CD8 + T-cell epitope
**SATC**	Ac-AAVVLLLWAAYKVAELVHFL	2268	81	CD8 + T-cell epitope with self-assembly properties
**TH**	Ac-AKFVAAWTLKA	1205	76	CD4 + T-cell epitope
**SATH**	Ac-AAVVLLLWKFERQAKFVAAWTLKA	2808	69	CD4 + T-cell epitope with self-assembly properties

#### Peptide synthesis, purification, and detection

The TC and TH peptides were synthesized based on the standard protocols of solid-phase peptide synthesis [27, 28]. The synthesized peptides were purified in preparative RP-HPLC using a C18 column (Knauer, Germany). The peptide masses were confirmed with High-resolution LC–MS Triple Quad 6410 (Agilent, Japan) instrument using the ESI method in positive mode. The [m + H]^+^ peaks of TC and TH appeared at 1055 and 1205, respectively, a similar value to that predicted for those peptides. Solid phase synthesized SATC and SATH peptides were purchased from ChinaPeptides (Shanghai, China). The supplier analysis sheet indicated > 90% purity, as determined by RP-HPLC using a C18 column.

To determine peptide contents of the purchased peptide powders, 1 mg of each peptide powder was introduced to an Elemental Analyzer (Eager 300 for EA1112, USA) and burned in pure oxygen to produce combustion products of CO_2_, H_2_O, and N_2_. Net weights of each peptide in the powder (Table 1) were calculated using the following equations [29]:

$$\text{Net } \text{peptide } \text{Content}=\frac{\mathrm{\%\text{N }} \text{obtained } \text{by } \text{CHN}}{\text{theoretical } \mathrm{\%N}}$$

$$\text{Pure peptide weight}=\text{Gross Weight}*\text{Net Peptide Content }(\mathrm{\%})*\text{HPLC purity}$$

The difference between gross and net weight might be due to the presence of humidity or salts.

#### Preparation and characterization of peptide nanoparticles

##### Preparation of peptide epitope nanoparticles

Peptide epitope nanoparticles were prepared by an optimized solvent displacement method. In this method, organic phase was an ethanolic solution of SATC peptide or the mixture of SATC and SATH peptides; hereinafter we called it SATC/TH. For ethanolic solution of SATC/TH, the mass ratio between the two peptides was set to be 6 SATC/ 1 SATH. The optimization was carried out on the protocol regarding the ethanol percentage, the mixing method, the stirring speed, and duration of the stirring (Fig. S2). Next, a series of F127 concentrations were prepared (Fig. S3). Upon optimization, four different nanoformulations listed in Table 3, with two different peptide compositions and two different F127 concentrations were prepared as follows. Briefly, 450 μl of aqueous F127 solution (5.5 mg/ml or 1.375 mg/ml F127 to achieve a 0.20 mM and or 0.05 mM F127 concentration in the formulation, respectively) was added dropwise to an HPLC vial containing 50 μl ethanolic peptide (SATC or SATC/TH) solution (10 mg/ml) under 500 rpm stirring for 30 min. Then, 500 μl PBS 10 mM was added dropwise to the sample, and stirring was continued for 30 min. The final concentration of the peptide in the nanoformulations was kept at 0.5 mg/ml. The prepared nanoformulations were immediately used for further analysis.

##### Critical aggregation concentration determination

The critical aggregation concentration (CAC) of the self-assembling peptide epitopes was determined using a Nile red assay*.* Different dilutions of nanoparticles in PBS (0.2–200 μg/ml) were prepared from the stock solution (0.5 mg/ml). A Nile red solution (0.2 µl, 1.25 mM in acetone) was added to 200 µl of each dilution. After overnight incubation at room temperature, fluorescence emission (Ex550/Em635) was measured by a synergy H4 ELISA reader (Biotek, Winooski, US) [30]. To determine the critical aggregation concentration (CAC), the fluorescence intensity at 635 nm was plotted versus the logarithm of peptide concentrations and the intersection was considered the CAC value.

##### Particle size and zeta potential measurement

The hydrodynamic diameter and polydispersity index (PDI) of the nanoparticles were analyzed using photon correlation spectroscopy (Zetasizer Nano-ZSTM, Malvern Instruments, UK). Zeta potential was determined by Laser Doppler Anemometry (Zetasizer Nano-ZS, Malvern Instruments, UK), measuring the mean electrophoretic mobility after 10X dilution of the nanoparticles in KCl 1 mM. All the analyses were performed in three replicates with a detection angle of 173° at 25 °C.

Nanoparticle tracking analysis (NTA) was performed to confirm particle size and nanoparticle concentration (Malvern Panalytical Ltd., Malvern, UK). For the analysis, nanoparticle samples were diluted 100X in ultrapure water. Each sample analysis was measured five times for 60 s using the Nanosight automatic analysis setting.

##### AF4 analysis method

Asymmetric Flow Field-flow Fractionation (AF4) analysis on different nanoformulations was performed with an AF2000 Multi Flow FFF instrument (Postnova Analytics, Landsberg, Germany) fitted with a series of detectors including RI (Refractive Index), UV and MALS (Multi-Angle Light Scattering). The fractionation was performed using a regenerated cellulose membrane (MWCO 10 kDa) and PBS 10 mM as carrier liquid. The channel flow was kept constant at 0.5 ml/min throughout the fractionation. The cross-flow was kept at 2 ml/min during the focusing step (5 min), the transition step (1 min) and the first 5 min of the fractionation. It was then lowered to 0.25 ml/min in 5 min, then to 0.1 ml/min in 5 min and finally to 0 ml/min in other 10 min. The cross-flow was then kept at 0 ml/min for further 15 min, to wash the channel and to ensure the complete elution of the sample (Fig. S4a). The formulations were analyzed as prepared without any further dilution or manipulation. The fractogram recorded by the UV detector at 280 nm was applied to examine the nanoparticles (Fig. S4b). The amount of free F127 in each sample was determined building a calibration curve with F127 standards of known concentration.

##### Circular dichroism

Circular dichroism spectra of the nanoparticles in PBS (at a concentration of 0.22 mM) were obtained by a double beam CD spectrometer (JASSCO with the model J-1100) using quartz cuvettes. Six measurements at a wavelength range from 180–250 nm were performed using 1 nm intervals at 25 °C. Then, the spectrum of the PBS was subtracted from the averaged spectrum of the samples.

##### Field emission scanning electron microscopy

For field emission scanning electron microscopy (FESEM) studies (ZEISS, Germany), the nanoparticles were diluted 10000X in water. Ten microliters of this mixture were placed on silicon wafers for 5 min and then washed with 1ml ultrapure water. The samples were left to dry at RT. Iridium sputtering was performed on dried samples for 10 min to make a 10 nm Iridium layer on the sample surface. The samples were observed on the microscope using InLens detectors, keeping the working distance (WD) at 2.3 mm and the extra high tension (EHT) at 3.00 kV.

##### Freeze-drying studies

A 500 µl trehalose solution (10% w/v) was added to 500 µl of the nanoparticles in a 5 ml freeze-drying glass vial and shaken horizontally for at least 10 min. The vials were quickly frozen in liquid nitrogen and then transferred to a -80 °C freezer at least 12 h before starting the 50 h freeze-drying process (Genesis 25 EL, S.P Industries, USA). First, samples were left in the freeze-drier at − 40 °C and a 200 mTorr vacuum for 4 h to guarantee that samples are completely frozen. The first drying phase was done at a temperature ranging from − 40 to + 20 °C, increasing the vacuum gradually from 200 to 20 mTorr for a period of 43 h. In the final step, the secondary drying phase was carried out for 3 h at + 22 °C and 20 mTorr. The freeze-dried nanoparticles were stored at 4 °C, and at fixed time points, they were resuspended in ultra-pure water and their properties were measured by DLS according to “Particle size and zeta potential measurement” section.

##### Colloidal stability in culture medium

A stability study was performed in RPMI supplemented with 10% FBS. Nanoparticle samples were diluted 10X with RPMI + FBS and then incubated at 37 °C while they were shaken at 100 rpm using a constant-temperature shaker. The size of the nanoparticles in RPMI media was measured by DLS every 2 h up to 8 h, with the attenuator fixed at 7.

##### Hemolysis test

Hemolysis test was conducted according to a standardized protocol [31]. Fresh human blood samples from healthy volunteers were obtained from Organ and Blood Donation Agency (ADOS; Santiago de Compostela. Spain) following informed consent and appropriate permission from the Institutional Ethics Committee (Comité Ético de Investigación de Galicia, CEIC; nº 2014/543). The samples were stabilized with ethylenediamine tetraacetic acid (EDTA). Blood samples were centrifuged at 300 g for 5 min to remove serum from the blood. The remaining red blood cells (RBCs) were washed 4 times with sterile PBS (10 mM) and then diluted 2X with sterile PBS. To determine the toxicity of nanoparticles against RBCs, 0.8 ml of nanoparticles at different concentrations (2, 10 and 20 μg/ml) were added to 0.2 ml of RBCs suspension, taking PBS and Triton-X-100 as the negative and positive control, respectively. Samples were vortexed, and then incubated at room temperature for 2 h. The nanoparticles and intact RBCs were removed by centrifugation. The absorbance (A) of hemoglobin in the supernatant was measured with a microplate reader (Synergy H1 Hybrid Multi-Mode, BioTek, Winooski, US) at 550 nm [32]. The absorbance at 655 nm was set as the reference, and the hemolysis percentage was calculated as:

$$\mathrm{\%}\ \text{hemolysis}=\frac{\text{Sample } {\text{A}}_{550-655}-\text{Negative } \text{control } {\text{A}}_{550-655}}{\text{Positive } \text{control } {\mathrm{A}}_{550-655}-\text{Negative } \text{control } {\text{A}}_{550-655}}\times 100$$

#### In vitro study of peptide nanoparticles with primary human DCs

##### Human DC generation

The *buffy coats* of human blood samples were collected following the same protocols and permissions stated in “Hemolysis test” section. Peripheral blood mononuclear cells (PBMCs) were isolated by Ficoll density gradient centrifugation [33]. Briefly, blood samples were diluted 2X with PBS and gently deposited on a Ficoll gradient in a 1:2 (Ficoll: blood) volume ratio. Different blood components were separated by centrifugation (400 g, 30 min, room temperature (RT), without brake). After removing the upper phase containing the plasma, PBMCs were collected from the interface. After washing twice with PBS (300 g, 10 min, RT), PBMCs were resuspended in the culture medium. Monocytes were isolated by surface adhesion to the culture plates. For this, the PBMCs were re-suspended in R2 (RPMI-1640 containing 2% FBS) and then 10 ml of this suspension was added to a 75 cm^2^ flask (T75, Thermo Scientific TM Bio Lite) and incubated for 2 h at 37 °C. Then, the non-adherent cells were removed by washing 3 times with PBS, and monocytes were cultivated for 6 days in R10 media (RPMI-1640 completed with 10% FBS) containing GM-CSF and Il-4, both at 100 ng/ml, to induce differentiation of monocytes to immature DCs (iDCs). To generate mature DCs (mDCs), bacterial lipopolysaccharide (LPS) and IFN-γ were added to iDCs at 10 ng/ml and 100 U/ml concentrations, respectively. The cells were incubated for 48 h at 37 °C to obtain mDCs.

##### Toxicity of the nanoparticles on iDCs

Once iDCs were generated, they were harvested by washing with cold PBS (300 g, 10 min, RT) and then re-suspended in RPMI. The obtained iDCs were incubated for 24 h with the different nanoparticles and at different concentrations (2, 22, and 45 μg/ml). PBS was used as a negative control. After the incubation period, cells were harvested and washed with PBS (1800 rpm, 5 min, RT). The toxicity of the different nanoformulations was determined by the MTS assay using a commercial kit (CellTiter 96^®^ AQueous Non-Radioactive Cell Proliferation Assay). After 4 h incubation of the cells with 10 μL of MTS reagent, the absorbance at 490 nm (considering λ = 630 nm as the reference wavelength) was measured and the viability percentage was calculated as:

$$\%\ \text{DC viability}=\frac{\text{Sample } \text{A}_{490-630}}{\text{Negative control } {\text{A}}_{490-630}}\times 100$$

##### Human DCs phenotype analysis

iDCs were incubated with the different nanoparticles at a final concentration of 10 µM for 48 h. PBS was used as a negative control and a combination of LPS (10 ng/ml) and IFN-γ (100 U/ml) were used as the maturation cocktail (positive control). To analyze the iDC phenotype, cells were washed twice with PBS and stained with anti CD1a-FITC, anti CD83-APC, and anti CD80-PE antibodies for 30 min, at 4 °C, in darkness. After washing with PBS, the phenotype of these DCs was analyzed by flow cytometry (BD FACSCalibur, Becton Dickinson, San Jose, CA, US) [34] and levels of maturation markers were quantified. Data were analyzed using the Flowing software (Cell Imaging Core, Turku Centre for Biotechnology, Turku, Finland).

##### Lymphocyte activation capacity of iDCs pre-incubated with different nanoparticles

After incubation of iDCs with the different nanoparticles for 48 h (10 µM), cells were harvested, washed with PBS, and plated with allogeneic T cells. After 2 weeks’ incubation, cells were harvested, washed with PBS, and stained with fixed amounts of dye-conjugated antibodies, including, anti CD4-APC, anti CD25-PE, anti CD8a-APC, and anti CD28-PE antibodies [35]. Lymphocyte activation was analyzed by flow cytometry using a BD FACSCalibur cytometer (Becton Dickinson, San Jose, CA, US).

#### In vivo studies

##### Immunization of C57BL/6 mice

To evaluate the immune response against the SATC (0.20 mM F127) nanoparticles in vivo, C57BL/6 mice were divided in four groups. Those in the untreated group were injected by PBS. The rest were immunized subcutaneously with i. soluble peptide (100 µg), ii. soluble peptide mixed 1:1 with complete Freund's adjuvant (CFA) (Sigma-Aldrich) as positive control and iii. nanoparticles (100 µg). After 12 days, mice were administered with their corresponding booster dose and after another 7 days, the animals were sacrificed by CO_2_ inhalation. The peripheral blood was taken, and the serum was collected after centrifugation at 16000 g for 10 min and it was frozen at − 20 °C until use. The spleens were extracted to prepare the cell suspension of splenocytes in PBS. All the experimental procedures were approved by the internal ethical research and animal welfare committee (Comité de Ética de Experimentación Animal, CEEA, nº 15010/16/002).

##### Isolation and culture of splenocytes

To obtain single-cell suspensions, spleens were passed through a 70-mm por size filter (Corning cell strainer; Corning, Sigma Aldrich) and centrifuged over a Ficoll gradient (Histopaque^®^-1083) in 15 mL falcon tubes. The different components were separated by centrifugation (400 g 30 min, RT, without brake). The upper fraction was removed and the cells from the interface were collected with a pasteur pipette. The cells were diluted with RPMI to 5 ml for washing (400 g, 10 min, RT), counted and re-suspended in the R10 culture medium to achieve a concentration of 5*10^6^ cell/well.

##### Determination of serum IgG antibody level by indirect ELISA

The presence of IgG antibody response in mouse blood sera were determined using an in-house indirect ELISA method. Epitope concentration, dilution of sera and secondary antibody were optimized by 2X serial dilutions. Based on the optimization data, 96-well plates were coated with 10 µg/ml of TC peptide and then blocked for 1 h with 0.2% of Tween-20 in PBS (PBST) containing 1% BSA. After removal of the blocking solution, sera at 25X and 50X dilutions were added, incubated for 2 h at room temperature, washed with PBST, and then incubated for 2 h with alkaline phosphatase conjugated secondary antibody (Goat Anti-Mouse IgG H&L, Abcam ab97020) at 10000X and 20000X dilutions. Plates were washed followed by incubation with p-nitrophenol phosphate substrate solution (N2770-5SET) for 30 min at RT. The reactions were terminated by addition of NaOH 0.75 M and optical densities were read using an ELISA plate reader (Synergy H1 Hybrid Multi-Mode, BioTek, Winooski, US) at 405 nm.

##### Analysis of antigen-specific responses by IFN-γ quantification by ELISPOT

IFN-γ enzyme-linked immunospot (ELISPOT) assays (U-CyTech Biosciences, Utrecht, The Netherlands) were used to detect antigen specific responses in treated mice according to the manufacturer’s instructions. Briefly, flat bottom 96-well plates (Maxisorp F96, NUNC) were filled with the coating antibody (37 °C 3 h). After washing the plates with PBS containing 0.5% Tween^®^20 (PBS-T) (Sigma Aldrich), they were blocked with blocking solution supplied by the manufacturer (at least 1 h at 37 °C). After the blocking period, the solution was removed by decanting and 4 × 10^3^ splenocyte cells were added in triplicate and incubated for 24 h to allow the capture of IFN- γ. Supplemented RPMI (R10) and TC soluble peptide epitope (5 µM) were used as negative and positive controls, respectively. Cells were removed by vigorous shaking, adding 200 µl/well of cold water and incubating the plates in an ice / water bath for 10 min. The plates were washed twice with PBS and 6 times with PBS-T. Then the biotinylated secondary antibody was added in a volume of 100 µl. The plate was incubated for 1 h (37 °C) and then washed 8 times with PBS-T before the addition of GABA (gold-labelled anti-biotin antibodies) in a volume of 50 µL. The plate was again incubated for 1 h (37 °C), washed 8 times with PBST and then emptied by a firm shake. 35 µl of the Activator I/II solution was added to each well (uniformly) in a dark place and after 10 min the development of spots was monitored. Once the spots have developed, the Activator I/II was removed, the plate washed with distilled water several times and dried in darkness until analysis. The number of IFN-γ secreting cells as the spot-forming cells (SFCs) were counted manually in an inverted optical microscope (Nikon Eclipse TS100). The average number of spots counted upon incubation with R10 (i.e., background) was subtracted from the number of spots counted upon peptide stimulation, and data are represented as the number of SFC per 10^6^ cells.

#### Statistical analysis

Data were analyzed with GraphPad Prism version 8.0 (GraphPad Inc.). The numbers are expressed as the mean ± standard deviation (SD) and mean ± standard error (SE) for physicochemical characterization of the nanoparticles and in vitro/in vivo studies, respectively. P values of 0.05 or less were considered statistically significant. All the experiments were repeated at least three times, except as otherwise specified.

## Results and discussion

**Fig. 1:**
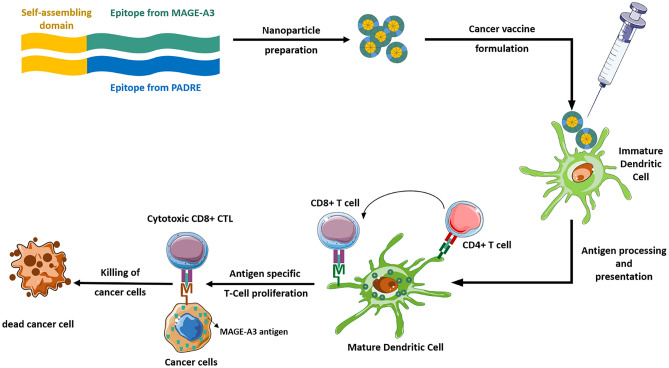
Scheme of the technological strategy: peptide diblocks consisting of PADRE/MAGE-A3 are self-assembled as nanoparticles. These immunoactive nanoparticles can interact with dendritic cells to activate CD4 + and CD8 + T-cells capable of killing cancer cells

In the present work, we aimed to design hybrid nanostructures consisting of peptide epitopes from MAGE-A3 antigen and PADRE attached to a self-assembling peptide as efficient systems capable of inducing CD8 + /CD4 + T cells responses (Fig. 1).

### Selection of the CD8^+^ and CD4^+^ T cell epitope sequences

The sequence (Ac-AAVVLLLW-COOH) is a self-assembly peptide domain (SA block) that has been previously reported [23]. The sequence KVAELVHFL (TC block) is a linear cytotoxic T-cell epitope (IEDB ID 33943) derived from MAGE-A3 (aa 112–120), which is a cancer-specific antigen present in 55% of the NSCLC patients [6]. A pan HLA-DR epitope (PADRE) with the sequence AKFVAAWTLKA was used as a universal helper T-cell epitope capable of binding a high number of MHC class II alleles (TH block) [36]. Peptides with a SA block at the N-terminus of both TC and TH epitopes were used to induce the formation of nanoparticles. Binding of epitopes to the transporters associated with antigen processing (TAP) is key for proper epitope presentation. AAY and KFERQ were used as linkers between SA and TC/TH segments, respectively. These sequences were selected aiming to generate C terminus epitopes suitable for binding to TAP and other chaperones [37, 38]. N-terminal of the peptides was acetylated to achieve a sequence with increased potential to enter the cells and degrade in the proteasome [39]. Therefore, the final designed sequences to induce CD8 + and CD4 + T-cells are Ac-AAVVLLLWAAYKVAELVHFL and Ac-AAVVLLLWKFERQAKFVAAWTLKA, referred here as SATC and SATH, respectively (Table 1).

In the IEDB server, the total score for the intrinsic potential of the peptides as T cell epitope is generated by combining predictors of proteasomal processing, TAP transport, and MHC binding. The binding affinity of SATC peptide to MHC-I was predicted using the IEDB analysis resource Consensus tool, which combines predictions from ANN aka NetMHC (4.0), SMM, and Comblib [40]. The IC 50 of TC to bind the HLA-A*02:01 human MHC-I allele, was 11 nM, well below 50 nM, the threshold for a high-affinity HLA-A*02:01 peptide epitope. The same prediction for the H2-K^b^ mouse MHC-I allele, showed again that TC has the best score among the computed epitopes, with an IC 50 of 241 nM. This value is below the 500 nM threshold and indicates an intermediate affinity for the H-2-k^b^ allele.

Even though these peptides consist of L-amino acids, experimental and theoretical studies suggest that they do not induce an adaptive immune response that interferes with our TC (KVAELVHFL) and TH (AKFVAAWTLKA) epitopes. A previous study using a peptide with the same self-assembly domain (AAVVLLLW) demonstrated that the immune response is specifically directed to the epitope domain and not the self-assembly domain [41]. Furthermore, the IEDB server's predictions for MHC I and MHC II binding affinity revealed that the SA (AAVVLLLW) sequence has a minimal probability of being a potential epitope for CD8 + or CD4 + T cells. The calculated IC50 values for SA binding to the HLA-A*02:01 human MHC-I allele and H2-Kb mouse MHC-I allele were 8115 nM and 22,705 nM, respectively. Both values exceed the threshold, indicating a low affinity of SA for these alleles.

The location of proteasomal cleavage for the SATC peptide on the antigen delivery pathway through MHC-I was analyzed in IEDB by using the MHC-NP tool, which predicts the naturally processed peptides by the MHC method. The predictions are based on in vitro proteasomal digests of the enolase and casein proteins [42]. The prediction data showed that in both human and mouse, TC sequence has the highest score among the other possible peptide segments for proteasomal cleavage through the MHC-I antigen-presenting pathway.

MHC-II binding predictions were performed by the IEDB analysis resource SMM-Align (ver. 1.1) [43]. The analysis showed that TH is a high-affinity peptide epitope for human MHC-II, with a predicted IC 50 for the binding between TH and HLA-DRB*01:01 of 49 nM. The prediction output for the H2-IAb allele of mouse MHC-II showed that TH has the best score among the computed epitopes. The estimated binding IC 50 was 980 nM, a value that classifies TH as a possible T-helper epitope for H2-IAb. Applying MHC II NP tool to predict the naturally processed MHC-II ligands of SATH, the TH sequence scored among the top 3 peptides regarding its possibility for cleavage through the MHC-II antigen-presenting pathway.

The allergenicity of the peptide sequence is one of the most important characteristics that need to be predicted before developing a vaccine formulation. The allergenicity of the designed peptide sequences was evaluated by AllerTOP, which is among the most accurate (88.7%) servers for this type of prediction [24]. Both SATC and SATH were found to be probably non-allergic. These peptides were also non-toxic as it was verified by using the ToxinPred module.

The theoretical isoelectric points (pI) of SATC and SATH were 5.1 and 10.93, respectively. The instability indexes of SATC and SATH were estimated to be 4.39 and 17.67, respectively, both below 40, which is indicative of stable peptides capable of initiating immune reactions. The computed aliphatic index for SATC and SATH were found to be 180.50 and 126.25, respectively, indicating that these sequences are thermostable. All peptides were insoluble in water and standard buffers. Exceptionally, SATC was soluble in basic aqueous solutions (NaOH 0.2M), but not SATH. Both peptides were soluble in hexafluoroisopropanol and ethanol. Due to its amenability for easy processing and better regulatory outlook, ethanol was used for nanoparticle preparation procedures.

### Characterization of peptides and pure peptide nanoparticles

The peptides that were used in this study and their characteristics are listed in Table 1. The CAC values of the SATC and SATC/TH were both ca. 6 μM, which is half of the value reported for SA2 [20] and indicate that the CAC in these formulations is dictated by the TC peptide sequence, as well as the self-assembly domain. On the other hand, the CAC value for SATH was 13.5 µM (data not shown), showing a minor impact of TH sequence on the CAC. This could be related to the higher hydrophilic/hydrophobic ratio in SATH as compared to SATC, due to the presence of the polar aminoacids threonine and glutamine in the SATH sequence. Addition of SATC to SATH decreased its CAC to levels indistinguishable from pure SATC (Table 2). Because of the hydrophobic nature of SATC and SATH and their limited solubility in buffered aqueous media, the corresponding nanoparticles were prepared using a solvent displacement method. This approach involved using ethanol as the solvent and water as the non-solvent (Table 2). The final pH of these formulations was acidic (Fig. S3d), and the protein nanoparticles showed a positive zeta potential more than 30 mV.

**Table 2:** Physicochemical characterization of the free peptide nanoparticles (mean ± S.D., n ≥ 3)

**Name**	**CAC (µM)**	**DLS size (nm)**	**PDI**	**pH**	**Zeta potential (mV)**
**SATC**	6.27 ± 0.21	129 ± 14	0.35	4.00 ± 0.12	38 ± 3
**SATC/TH**	6.41 ± 0.63	133 ± 19	0.25	3.55 ± 0.02	30 ± 4

**Fig. 2:**
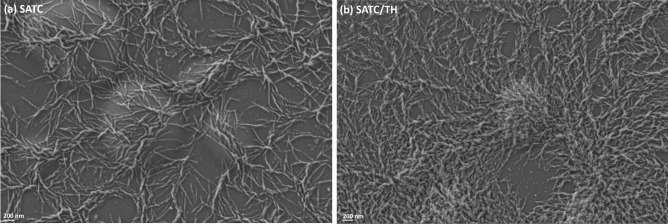
FESEM images of **a** SATC and **b** SATC/TH nanostructures at 10^6^X dilution

Peptide nanoparticles were stable when formed in ultrapure water. However, they underwent quick fibrillation in any buffered media simulating physiological conditions, as observed by FESEM imaging (Fig. 2). This observation was not unexpected since these peptides form β-sheet secondary structures in PBS (see “Physicochemical characterization of selected peptide nanoparticle compositions” section), and those tend to self-assemble into fibrils [23]. The immune response induced by fibrils does not significantly involve cellular immunity, and thus, it is critical to maintain a nanoparticulated granulometry to evoke a cellular immune response [22, 44, 45].

### Synthesis of stabilized self-assembled peptide nanoparticles

Stabilized nanoparticles from the self-assembling peptide epitopes were prepared by a solvent displacement method (Table 3, Supplementary Information Fig. S1).

**Table 3:** Physicochemical characterization of the F127 stabilized peptide nanoparticles used in this study (mean ± S.D., n ≥ 3)

**Name**	**CAC (µM)**	**DLS size (nm)**	**PDI**	**pH**
**SATC (0.05 mM F127)**	7.25 ± 0.08	157 ± 29	0.14	7.31 ± 0.02
**SATC (0.20 mM F127)**	7.54 ± 0.22	167 ± 43	0.17	7.30 ± 0.09
**SATC/TH (0.05 mM F127)**	6.78 ± 0.51	153 ± 32	0.15	6.83 ± 0.15
**SATC/TH (0.20 mM F127)**	7.52 ± 0.42	133 ± 32	0.12	7.03 ± 0.05

To control the self-assembly and fibrillation of the peptide nanoparticles, a stabilizing agent (poloxamer) was added to the external medium. Poloxamers have an amphiphilic nature, allowing them to form a sterically stabilizing, hydrated polyoxyethylene shell for nanostructures [46–50]. Besides its stabilization function, poloxamers are biocompatible and have other beneficial pharmaceutical properties, as cryoprotectants for lyophilization [47, 51–53] and as modulators of immune responses [54]. Herein, the peptide nanoparticles were stabilized with Pluronic F127 (Poloxamer 407; F127), and the presence of this pharmaceutical excipient prevented indefinite assembly of β-sheets to form fibrils. F127 has been previously utilized to stabilize poly(propylene sulfide) (PPS) nanoparticles [45] and self-assembling silk sericin protein nanoparticles [55], among others.

The DLS analysis of the nanoparticles with different F127 concentrations showed no difference in particle size and PDI (Fig. S3a and b). However, there is a significant increase in terms of count rate as we decrease the concentration of F127 in the formulation (Fig. S3c). This could be related to fibril formation in the absence of polymeric stabilizers, as suggested by FESEM images, where nanoformulations appear as elongated precipitate structures (see “Characterization of peptides and pure peptide nanoparticles” section). F127 concentrations in our peptide nanoparticles were 0.05 and 0.20 mM, which are below its CAC, 0.5 mM. This was important to prevent the formation of a second population of pure F127 micelles.

### Physicochemical characterization of selected peptide nanoparticle compositions

CAC determination showed effective assembly in nanoparticle preparations (Table 3). The CAC values of the peptides increased as we increased the concentration of F127, but they remained consistently low in all cases (< 8 µM; Table 3). Size determination of the nanoparticles by DLS showed that all the nanoparticles were smaller than 200 nm, which has been suggested to be the optimum range for efficient uptake by DCs, and for generating cellular immune responses [56–60]. PDI was always consistently low (< 0.2).

**Fig. 3:**
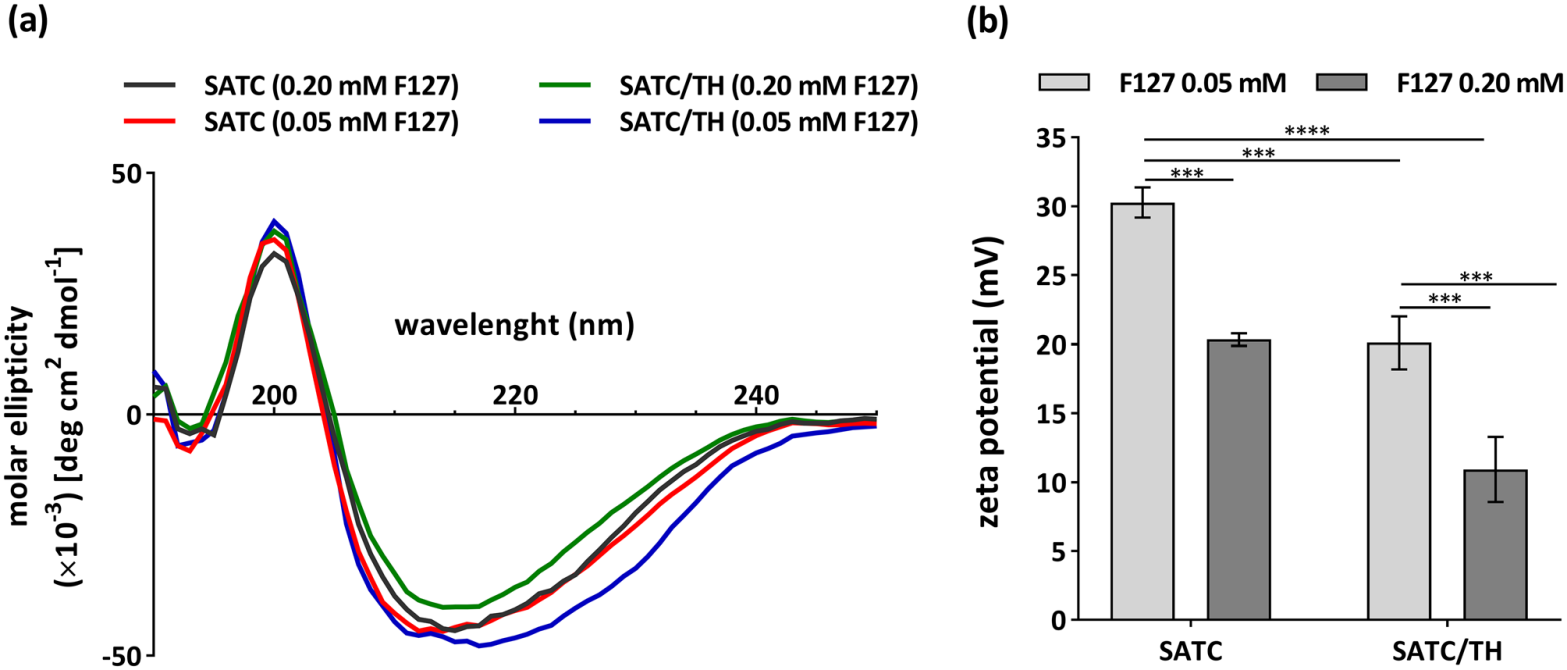
**a** CD spectra of the nanoparticles in PBS at 0.22 mM peptide concentration. **b** Zeta potential of the nanoparticles (mean ± S.D., n ≥ 3). Data were analyzed with one-way ANOVA, Tukey test, *P* < 0.05

To investigate the inner structure of the peptide nanoparticles, we performed circular dichroism analysis (Fig. 3a). The CD spectra of the nanoparticles in PBS had one strong minimum at 215 nm, indicating the formation of β-sheets within the structure of the peptide nanoparticles. This data confirms that self-assembly is directed by these secondary structures.

Unlike the free nanoparticles, the final pH of all the stabilized nanoformulations were neutral (Table 3). Protein nanoparticles presented a positive zeta potential, which has great importance for efficient uptake by DCs [58, 61]. The zeta potential values for all the nanoparticles were in the range between + 10 and + 30 mV (Fig. 3b), which suggest enough ionic repulsion to maintain the colloidal stability of the nanoformulations [62]. It has been shown that subcutaneous administration of positively charged nanoparticles with size larger than 100 nm might result in their capture by APCs and drainage to the lymph nodes [63]. These types of nanoparticles are assumed to stimulate cross-presentation of CD8 + T-cell responses through increasing the lysosomal pH in DCs and limiting antigen degradation [64]. The zeta potential of the nanoparticles decreased as the concentration of F127 was increased (Fig. 3b). This is a typical charge shielding effect related to the displacement of the nanoparticles shear plane, resulting from the formation of a hydrophilic polyoxyethylene shell. Besides, SATC/TH protein nanoparticles showed a lower zeta potential than pure SATC nanoparticles. This could be related to charge neutralization and higher number of electrostatic interactions between the residues with opposite charge in SATH and SATC in comparison to the formulations that only contain SATC peptide. Due to these two factors, the lowest zeta potential was observed for SATC/TH (0.20 mM F127).

**Fig. 4:**
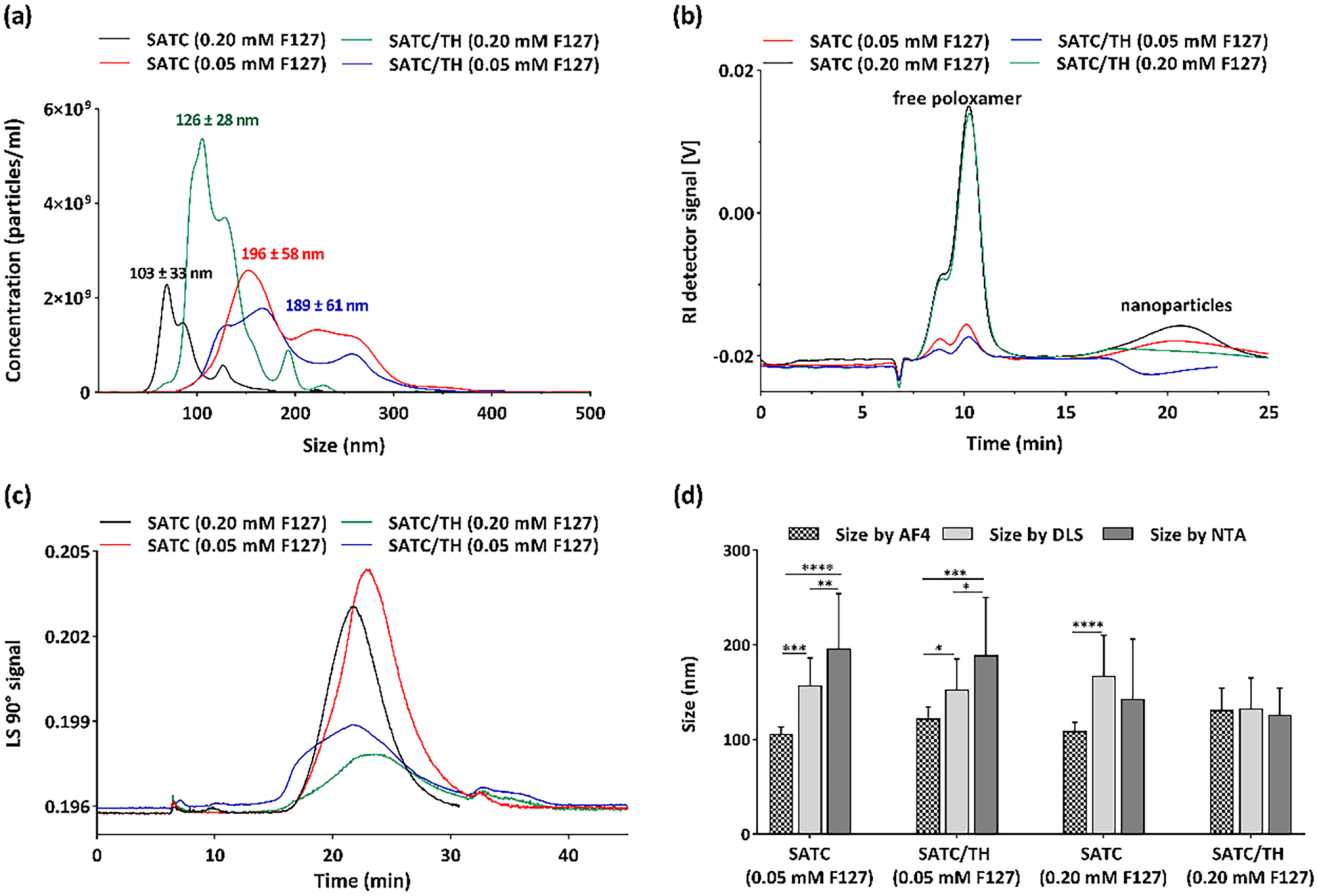
**a** Representative image of the particle size distribution obtained by nanoparticle tracking analysis (mean ± S.D.). **b** Fractograms of the nanoparticles recorded by RI detector. **c** Fractograms of different nanoparticles recorded by MALS detector. The signal of the blank (blank = 40 μL of MilliQ water) was subtracted to all the series. **d** Size of the nanoparticles measured by different methods (mean ± S.D., n ≥ 3). Data were analyzed with two-way ANOVA, Tukey test, *P* < 0.05

DLS size measurements were confirmed by NTA and AF4 (Fig. 4), following orthogonal analysis recommendations of EUNCL and NCI-NCL [65]. DLS correlate the average fluctuation in the intensity of the scattered light to the size of the nanoparticles. Since in NTA the scattered light of the individual nanoparticles is monitored, it provides more accurate and independent size measurements compared to DLS [62]. Nanoparticles with 0.05 mM F127 concentration showed more differences between these two techniques. The NTA size was approximately 40 nm lower than the DLS size for these peptide nanoparticles (Fig. 4a). These differences in size suggest that these formulations are less homogenous than the formulations with lower F127 concentration.

It is considered that the size distribution of nanoparticles determined by separative techniques of high resolution such as AF4 is more accurate than those obtained by techniques such as DLS [66]. Besides, AF4 is particularly interesting for the characterization of complex systems, where different species populations can be formed. Since unbound monomeric poloxamer could also be present in our samples, we characterized our nanoparticle suspension by this technique. The fractogram reported with the UV detector at 280 nm indicated peaks at 5–10 min and 20–27 min (Fig. S4b). The first one is the void peak formed by non-separated particles. The second peak corresponds to the nanoparticles. The free poloxamer cannot be detected by the UV spectrometer, and no further populations were identified in this UV fractogram. The small hump at 35–37 min was attributed to the release of a small quantity of larger particles when the fractionation is interrupted. The fractogram recorded with the refraction index detector could be used to quantify the free poloxamer (Fig. 4b). Between 8–12 min two peaks corresponding to free poloxamer were observed. The area of the second peak at 9–12 min was used to build the calibration curve between F127 sample concentration and the observed signal, as it resulted in the best linearity. By applying this calibration curve to the peaks observed in the formulations, we could calculate that the free poloxamer for all the formulations was around 80% of the total. The fractogram recorded by the MALS detector only indicated one major peak that could be assigned to the peptide nanoparticles (Fig. 4c). This fractogram with the MALS detector could also be used for an absolute determination of particle size distribution based on the angular dependence of the light scattered. For this sample, a random coil model was used to determine the radius of the nanoparticles. In general, all the particle sizes data measured by the three techniques stayed within a narrow range. The best agreement among these three techniques was observed for SATC/TH (0.2 mM F127) nanoparticles, with less than a 10 nm difference (Fig. 4d).

**Fig. 5:**
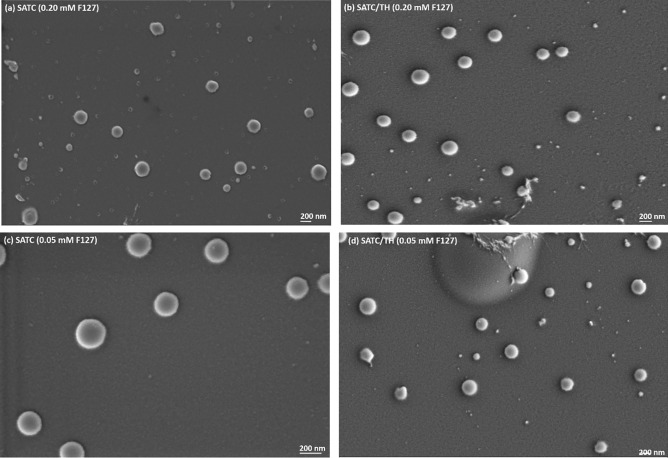
FESEM images of **a** SATC (0.20 mM F127), **b** SATC/TH (0.20 mM F127), **c** SATC (0.05 mM F127) and **d** SATC/TH (0.05 mM F127)

FESEM images showed the spherical morphology of the nanoparticles and supported the data obtained by AF4, DLS and NTA regarding the general particle size range (Fig. 5).

### Cell culture medium and blood-nanoparticle compatibility

**Fig. 6:**
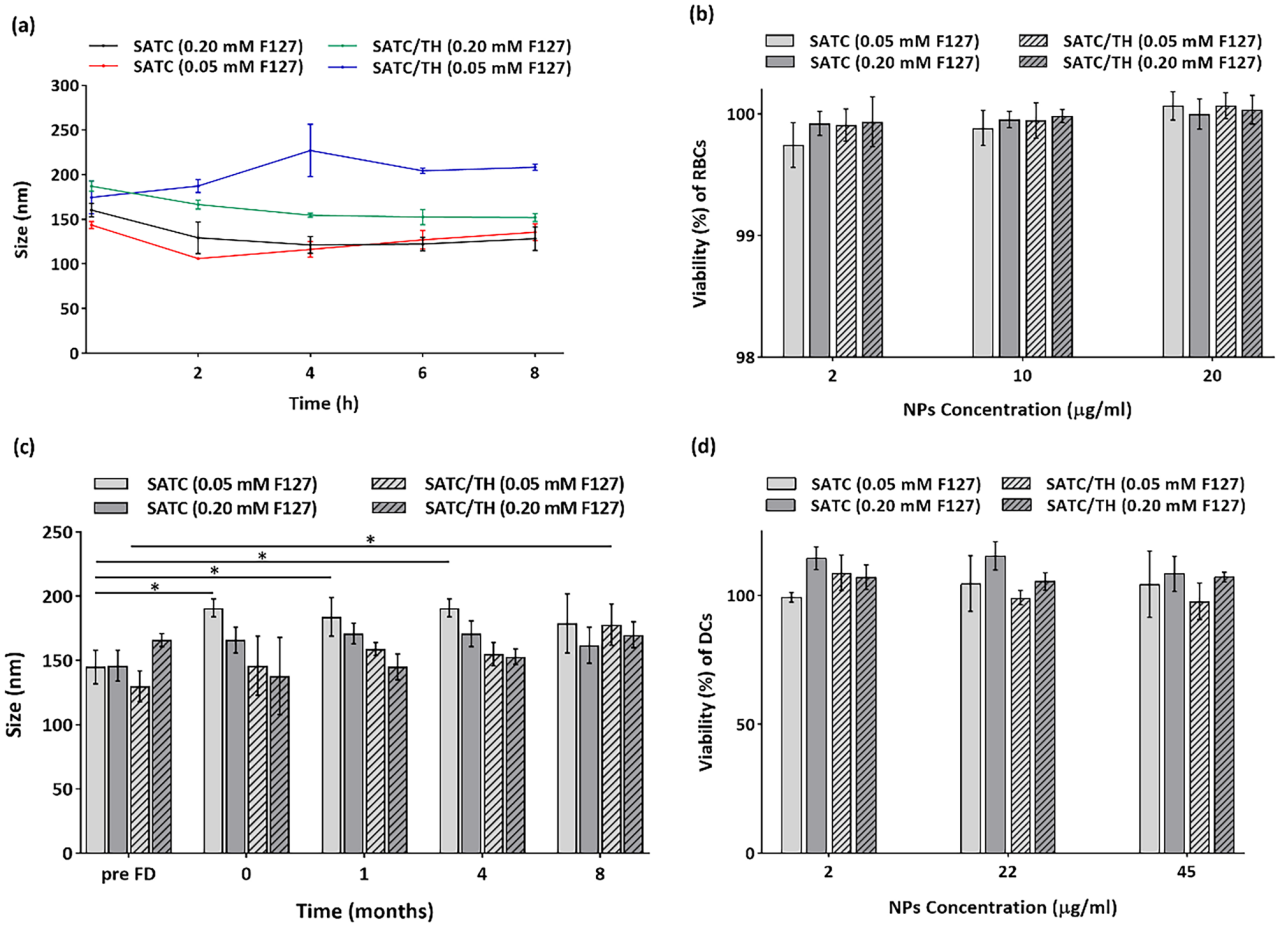
**a** Size stability of the nanoparticles during 8 h incubation in RPMI supplemented with 10% FBS at 37 °C (Mean ± S.E., n = 3). **b** Viability of RBCs after 2 h incubation with the Nanoparticles (mean ± S.E., n = 4). **c** Particle size evolution of the freeze-dried nanoparticles upon storage at 4 °C (mean ± S.D., n = 3). All formulations were freeze-dried with trehalose. **d** Viability of DCs after 24h incubation with nanoparticles (mean ± S.E., n = 4). All Data were analyzed with two-way ANOVA, Tukey test, *P* < 0.05

Nanoparticle stability in culture media is essential to ensure that their pharmaceutical properties are maintained for cell experiments. Also, since RMPI is a neutral pH buffer, a stability study in this medium would provide information on how the system might behave upon injection. Therefore, the size of the nanoparticles was monitored for 8 h (RPMI supplemented with FBS at 10%, 37 °C). No significant changes in the main parameters characterizing the particle size distribution were observed during this period: average particle size (Fig. 6a), count-rate and PDI were all constant (Fig. S5a and b). This result suggests that these poloxamer-coated protein nanoparticles have adequate colloidal stability in cell culture and buffered media.

Since all the nanoparticles, regardless of administration route, would pass the blood to reach their target tissues, it is necessary to evaluate nanoparticles-erythrocyte compatibility [67]. To check this, we performed a test where the release of haemoglobin into the plasma is measured as a readout for erythrocyte lysis. After 2h incubation of the peptide nanoparticles with red blood cells, we observed no haemoglobin release, even at the highest concentration tested (20 µg/ml). This result indicated that the peptide nanoparticles are highly compatible with blood cells (Fig. 6b), and the overall results suggest the suitability of these peptide nanoparticle formulations for IV administration.

### Storage stability

Long-term stability during storage conditions is an essential feature for the pharmaceutical development of vaccine formulations. In our case, we checked the possibilities for freeze-drying our nanovaccines both by themselves, and in the presence of a cryoprotectant. We selected trehalose as a cryoprotectant because it has low hygroscopicity and chemical reactivity, capacity to form hydrogen bonds, and a high glass transition temperature [68–70]. Freeze-drying in the absence of the trehalose resulted in aggregates that could not be redispersed. By adding 5% trehalose, the freeze-dried cakes became completely redispersable. The DLS analysis of the nanoparticles after freeze-drying showed that the nanoparticles maintain their size and PDI, except for SATC (0.05 mM F127), where particle size increased significantly, around 50 nm (Fig. 6c). In general, this data indicates a successful freeze-drying process, and this presentation could be used as the final vaccine dosage form, to provide better stability.

### In vitro study of the interaction of peptide nanoparticles with primary human DCs: cytotoxicity

DCs, are professional APCs broadly distributed throughout the body and capable of recognizing multiple molecular structures present in pathogens [71]. As the only skilled cells of the immune system at cross-presentation, they can capture, process, and present exogenous antigens. DCs can migrate from peripheral tissues to lymphoid tissues, where they activate naïve lymphocytes and direct them towards differentiation into effector cells [72]. These characteristics make DCs key players for producing an immune response against various antigens. Because of the importance of DCs activation, we studied the interaction of peptide nanoparticles and DCs in vitro, as the first measure of their potential as a cancer vaccine.

DCs could be generated from PBMCs following the treatment with GM-CSF and interleukin 4(IL-4) [73]. The non-toxic nature of the peptide sequences was confirmed by ToxinPred module (“Selection of the CD8+ and CD4+ T cell epitope sequences” section). To prevent unspecific effects, we first analysed the cytotoxicity of peptide nanoparticles against dendritic cells was evaluated by MTS assay, for 24 h, and at three nanoparticle concentrations (Fig. 6d). The nanoparticles showed no cytotoxicity even at the highest concentration tested (45 µg/ml). The results indicate that these peptide sequences are highly cell compatible, even in nanoparticulated form. This compatibility would be the result from sequences with low amounts of cationic residues, since it has been previously shown that soluble cationic substances show higher cell adhesion properties [74]. These results imply that final combination of the size, zeta potential and concentration of these peptide nanoparticles are within the compatibility range for the cells.

### Change in iDCs phenotype

**Fig. 7:**
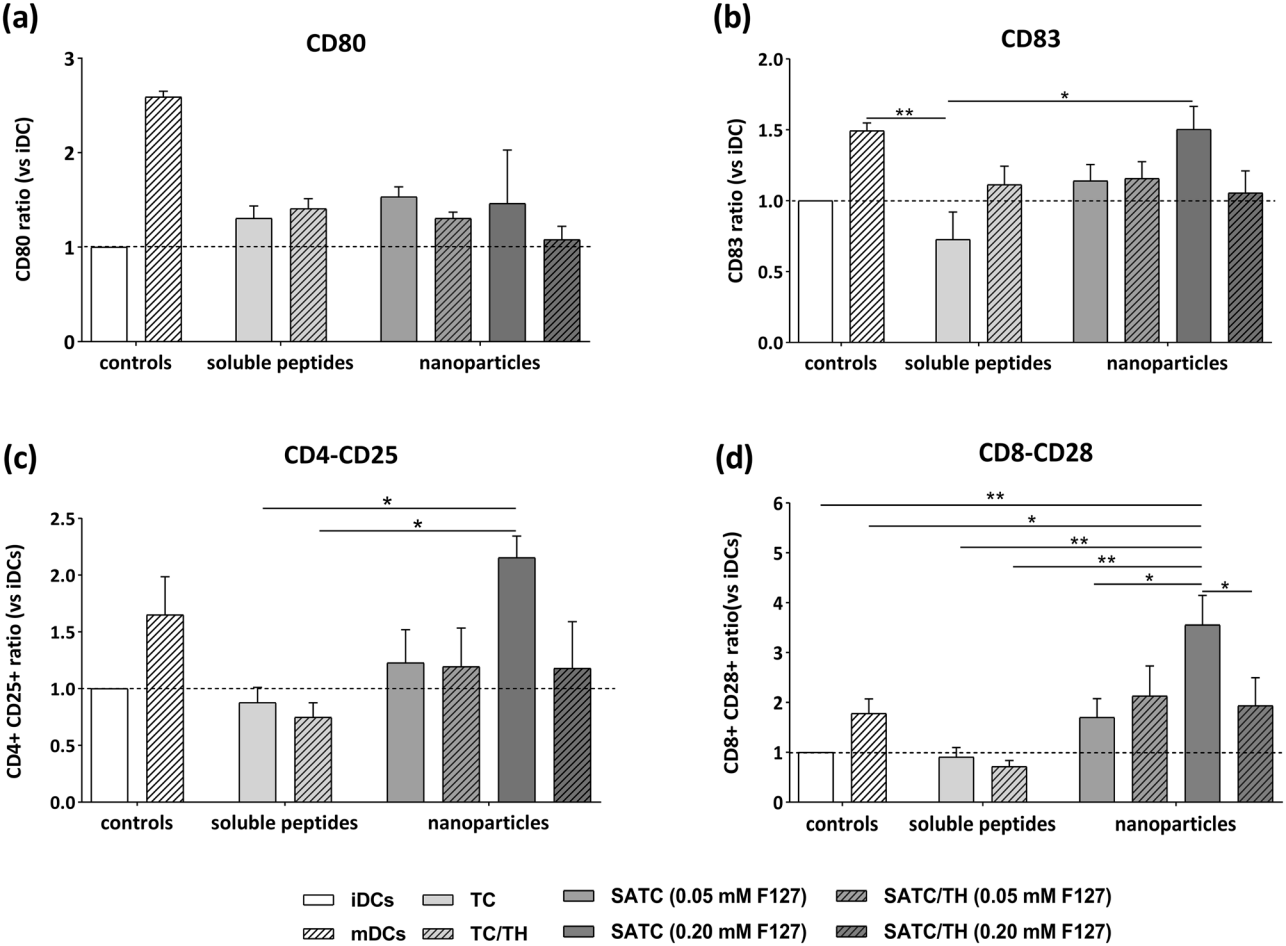
In vitro immune activation studies. **a** CD80 + and **b** CD83 + dendritic cell phenotype by incubation of iDCs with nanoparticles at 10 µM for 48 h. Data are shown as the ratio (%) between the mean fluorescence intensity (MFI) of the corresponding marker in iDCs incubated with the different nanoparticles versus the MFI of iDC incubated in culture media (mean ± S.E., n = 4). Data were analyzed with one-way ANOVA, Sidak test, *P* < 0.05. The capacity of the iDCs treated by nanoparticles to activate allogeneic CD4 + (**c**) and CD8 + (**d**) T lymphocytes was determined by flow cytometry and quantifying the upregulation of CD25 (**c**) and CD28 (**d**), respectively. Results are shown as the ratio between the total event number of CD4 + CD25 + (CD8 + CD28 + T) cells using iDCs incubated with nanoparticles versus total event number of CD4 + CD25 + (CD8 + CD28 +) T cells using iDCs incubated in culture media (mean ± S.E., n = 4). Data were analyzed with one-way ANOVA, Tukey test, *P* < 0.05

The process of T cell proliferation and differentiation involve signals provided by mature DCs. Phenotypic maturation of DCs upon antigen uptake is associated with upregulation of the costimulatory molecules CD80 and CD83 (Fig. S6a) [75]. To evaluate the effect that different peptide nanoparticles may have on iDCs phenotype and functionality, the expression of these markers were analyzed by flow cytometry. The two peptide sequences without the self-assembling domain, TC and TC/TH, were used as controls. TC and its combination with TH are soluble peptides and do not form nanoparticles. To achieve a more accurate quantification of the monocytes differentiation, CD1a was selected as a DC marker. Analysis of DCs phenotype demonstrated that iDCs incubated with peptide nanoparticles showed a tendency for the upregulation of CD80 and CD83 activation markers, but without reaching statistical significance (Figs. 7a and b, and S6b). Indeed, most nanovaccine groups showed markers that were not significantly different from neither the negative (iDC) nor the positive (mDC) controls. Even without a clear indication of DCs phenotype shift, it is possible that these cells could affect T lymphocyte activation. We checked this possibility as a next step.

### Stimulation of CD8^+^ and CD4^+^ T lymphocytes

During T lymphocyte activation, CD28, a T cell ligand from the B7 family, is expressed on the surface of the mature DCs providing the necessary co-stimulation to these cells. Interleukin-2 (IL-2) cytokine, mainly secreted by CD4 + T lymphocytes, binds to CD25, the high-affinity IL-2 receptor expressed on the surface of an antigen-stimulated CD4 + T cell, during their late-stage proliferation and differentiation [76]. We performed a flow cytometry analysis to assess the capacity of iDCs, previously stimulated by peptide nanoparticles, to act as activators of allogeneic CD8 + and CD4 + T lymphocytes (Fig. 7c and d). The results were compared to cells activated with non-stimulated iDCs and iDCs stimulated with TC/TH (peptides without the self-assembly domain). The results showed that pre-incubation of iDCs for 48 h with the peptide nanoparticles at 10 µM, could increase the number of activated CD4 + T lymphocytes (determined as CD4 + CD25 + cells) (Fig. 7c). Besides, all the peptide nanoparticles demonstrated an increase in activated CD8 + T cell numbers (determined as CD8 + CD28 + cells) compared to free peptide controls. On the other hand, iDCs primed with the soluble peptides, TC and TH, were unable to activate CD8 + T cells (Fig. 7d). This could be related to enhance cellular uptake of the particulate systems, and the high density of antigenic epitopes on the surface of the nanoparticles. These results are also in agreement with previous studies where other peptide epitopes were extended by incorporating a self-assembling domain [77], which suggest that this might be a general approach to mount enhanced immune responses by reducing T cell anergy [23]. Since the nanoparticles of SATC (0.20 mM F127) showed higher levels for CD83, CD4 + /CD25 + and CD8 + /CD28 + T cells activation in comparison to other nano-formulations, it was selected to perform the in vivo studies. The better performance of these nanoparticles in stimulating T lymphocytes in vitro could be related to a more efficient uptake and cross-presentation by DCs, as these nanoparticles contain the higher concentration of F127 stabilizing poloxamer (0.20 mM) along with a relatively higher positive zeta potential compared to the other formulations [64].

### In vivo humoral and cellular responses

**Fig. 8:**
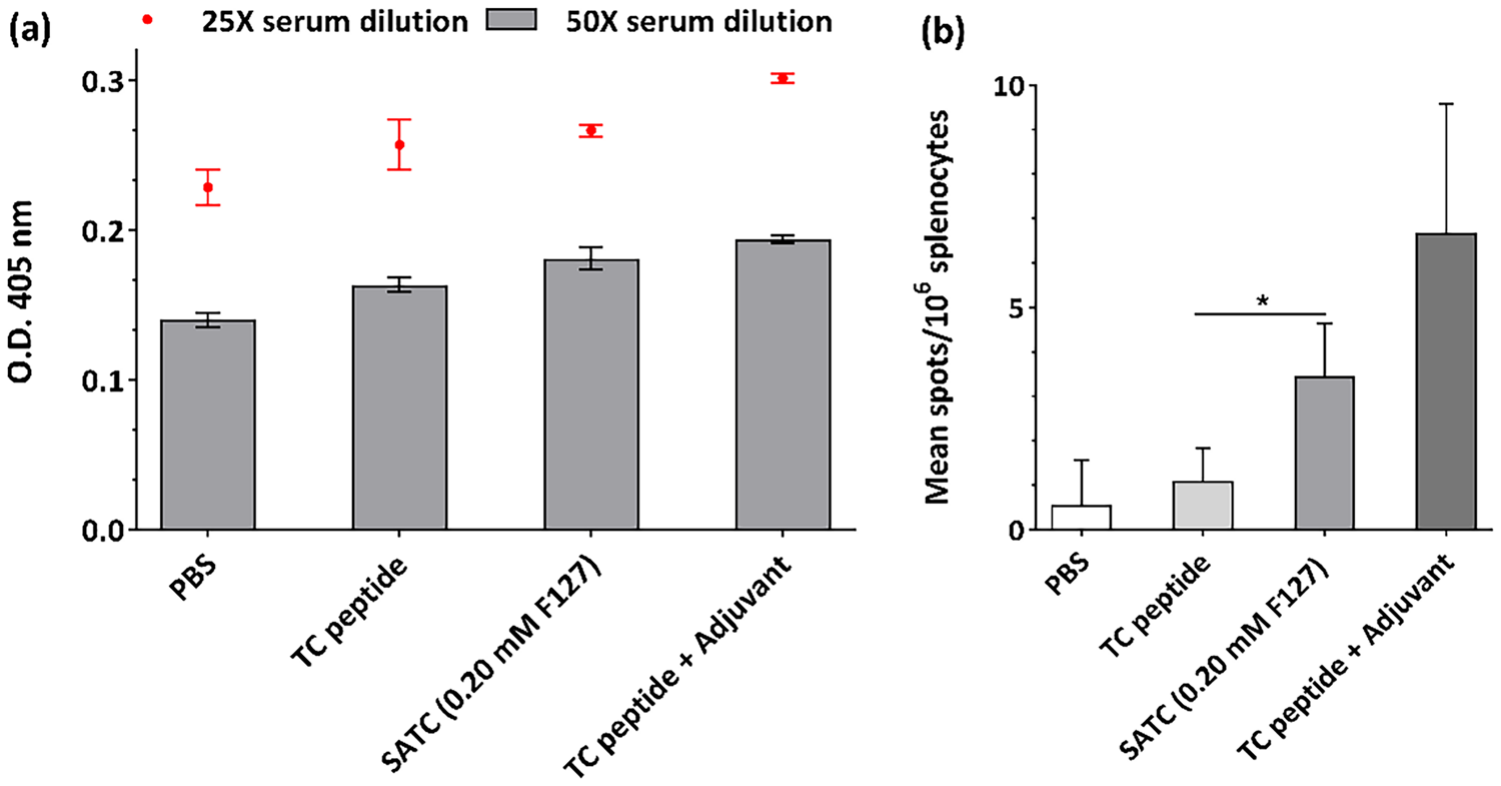
In vivo immune activation studies. **a** Optical densities (λ = 405 nm) observed at 25X and 50X mice sera dilutions one week after receiving booster doses at 10000X dilution of the secondary antibody. Data were analyzed with two-way ANOVA, Tukey test, *P* < 0.05 (mean ± S.E., n = 3 mice per group). All groups were statistically different from PBS and equal among them. **b** IFN-γ production was determined by ELISPOT assay in response to stimulation with TC epitope. Data are expressed as spots/10^6^ splenocytes ± S.E. for each group (n = 3). A t-test analysis was performed comparing the values of immunized mice versus naïve (non-immunized) mice (*P* < 0.05)

Based on the previous data, an in vivo study was performed where the peptide nanoparticles were administered to C57BL/6 mice in a prime-boost regimen. The treatment group received nanoparticles of SATC (0.20 mM F127). One group received soluble peptide without a self-assembling domain, TC peptide, and the positive control group was vaccinated with TC peptide associated to Freund's adjuvant. In the IgG-ELISA analysis, treated mice groups showed significant levels of IgG antibody responses in comparison to the negative control groups, but equal among themselves (Figs. 8a and S7). This IgG antibody response suggests a CD4 + Th-cell dependent antibody class switching mechanism. If the peptide nanoparticles are taken and processed by lysosomes and late endosomes they will undergo a class II MHC pathway, and their epitopes are presented to CD4 + T cells. The results agree with the in vitro CD4 + lymphocyte stimulation results, where we observed an elevated number of activated CD4 + T-lymphocytes (CD4 + CD25 +) for nanoparticles of SATC (0.20 mM F127; Fig. 7c). Studies on the cytokines related with CD4 + activation, such as IL4, could help to understand better the activation mechanisms, and whether this antibody production is T cell dependent or not.

IFN-γ ELISPOT is a sensitive assay to quantify the level of cellular immune response, since CD8 + and CD4 + effector T cells and activated DCs produce IFN-γ. Subcutaneous immunization of C57Bl/6 mice followed by a booster dose on day 12 with the nanoparticles of SATC (0.20 mM F127) induced activated specific T cells that secreted IFN-γ, while soluble peptide groups failed to induce any significant T cell responses in mice (Fig. 8b). These results confirm the in vitro results on antigen presentation by DCs, where self-assembling peptide nanoparticles could efficiently activate DCs. As mentioned earlier, activated DCs produce IFN-γ, which initiate the T cell-DC crosstalk and thereby elicit T-cell priming [78]. The lack of IFN-γ response detected in the soluble TC peptide group (no self-assembly domain) could be caused by a transient effector CD8 response, rather than by a memory response, as previously reported by other studies [79, 80]. These results agree with other studies reporting limited uptake of soluble peptides by DCs [13]. Moreover, previous works have shown significant degradation of soluble peptides by proteolytic peptidases on the surface of DCs [81]. F127 in the nanoparticles of SATC (0.20 mM F127) could protect peptide epitopes against protease degradation by providing a prolonged antigen presentation to APCs through peptide epitope exposed to the immune system. The results of IgG-ELISA and IFN γ-ELISPOT indicates that both class II and class I MHC pathways are involved in the immune response against the TC epitope. However, the MHC I pathway is the most dominant immune pathway in the case of SATC nanoparticles compared to the TC soluble peptide epitopes.

Overall, our results are in consistent with previous approaches using self-assembling peptide epitopes in their particulate form [80, 82, 83]. In these studies, long peptide sequences were used to drive the self-assembly process that trigger uncontrolled immune reactions. Although there are various studies on development of self-assembled peptide-based vaccines to raise cellular response against cancer, most of them employ self-assembling peptides only as drug delivery vectors. The main nanostructure in these studies are nanofibers, which are less effective than a particulate structure to stimulate the cellular immune response. In this study, we took advantage of a nanoparticulated antigen by co-assembly of self-assembling peptide epitopes with poloxamer. This would help us to overcome the limitations related to the use of long peptide sequences and fibrous structures.

## Conclusion

Stable nanoparticles can be formed from engineered self-assembling peptide antigenic sequences. These structures are suitable as injectable nanovaccines when stabilized with poloxamer. These nanoparticles can activate DCs and effector cells and are able to elicit a strong cellular response in vivo. On the contrary, the peptide antigens lacking the self-assembling sequence induce sub-optimal responses. This data supports the interest of these peptide nanoparticles as a new therapeutic nanovaccine platform.

### Supplementary Information

Below is the link to the electronic supplementary material.

Supplementary file1 (DOCX 795 kb)

## Data Availability

The datasets generated during and/or analysed during the current study are available from the corresponding author on reasonable request.
