# Supplementary material for: Self-assembled peptide/polymer hybrid nanoplatform for cancer immunostimulating therapies
Source: Drug Deliv Transl Res. 2023 Sep 18;14(2):455–73. doi: 10.1007/s13346-023-01410-y (PMC10761384; doi:10.1007/s13346-023-01410-y)
Supplement: Supplementary file 1 — Supplementary file1 (DOCX 795 kb) [file 13346_2023_1410_MOESM1_ESM.docx]

# Self-assembled peptide/polymer hybrid nanoplatform for cancer immunostimulating therapies

**Drug Delivery and Translational Research**

**Supplementary material**

Saeedeh Khazaei^1,2^, Ruben Varela-Calviño^3^, Mazda Rad-Malekshahi^1^, Federico Quattrini^2^, Safura Jokar^4^, Nima Rezaei^5^, Saeed Balalaie^6^, Ismaeil Haririan^1,*^, Noemi Csaba^2^, Marcos Garcia-Fuentes^2,*^

*^1^ Department of Pharmaceutical Biomaterials and Medical Biomaterials Research center, Faculty of Pharmacy, Tehran University of Medical Sciences, Tehran, Iran.*

*^2^ Department of Pharmacology, Pharmacy and Pharmaceutical Technology, CiMUS Research Center and Health Research Institute of Santiago de Compostela (IDIS), University of Santiago de Compostela, Santiago de Compostela, Spain.*

*^3^ Department of Biochemistry and Molecular Biology, School of Pharmacy, University of Santiago de Compostela, Spain.*

*^4^ Department of Nuclear Pharmacy, Faculty of Pharmacy, Tehran University of Medical Sciences, Tehran, Iran.*

*^5^* *Department of Immunology, School of Medicine, Tehran University of Medical Sciences, Tehran, Iran.*

^6^ *Peptide Chemistry Research Center, K. N. Toosi University of Technology, Tehran, Iran.*

**correspondence to:* [*marcos.garcia@usc.es*](mailto:marcos.garcia@usc.es) *(MGF), haririan@tums.ac.ir*


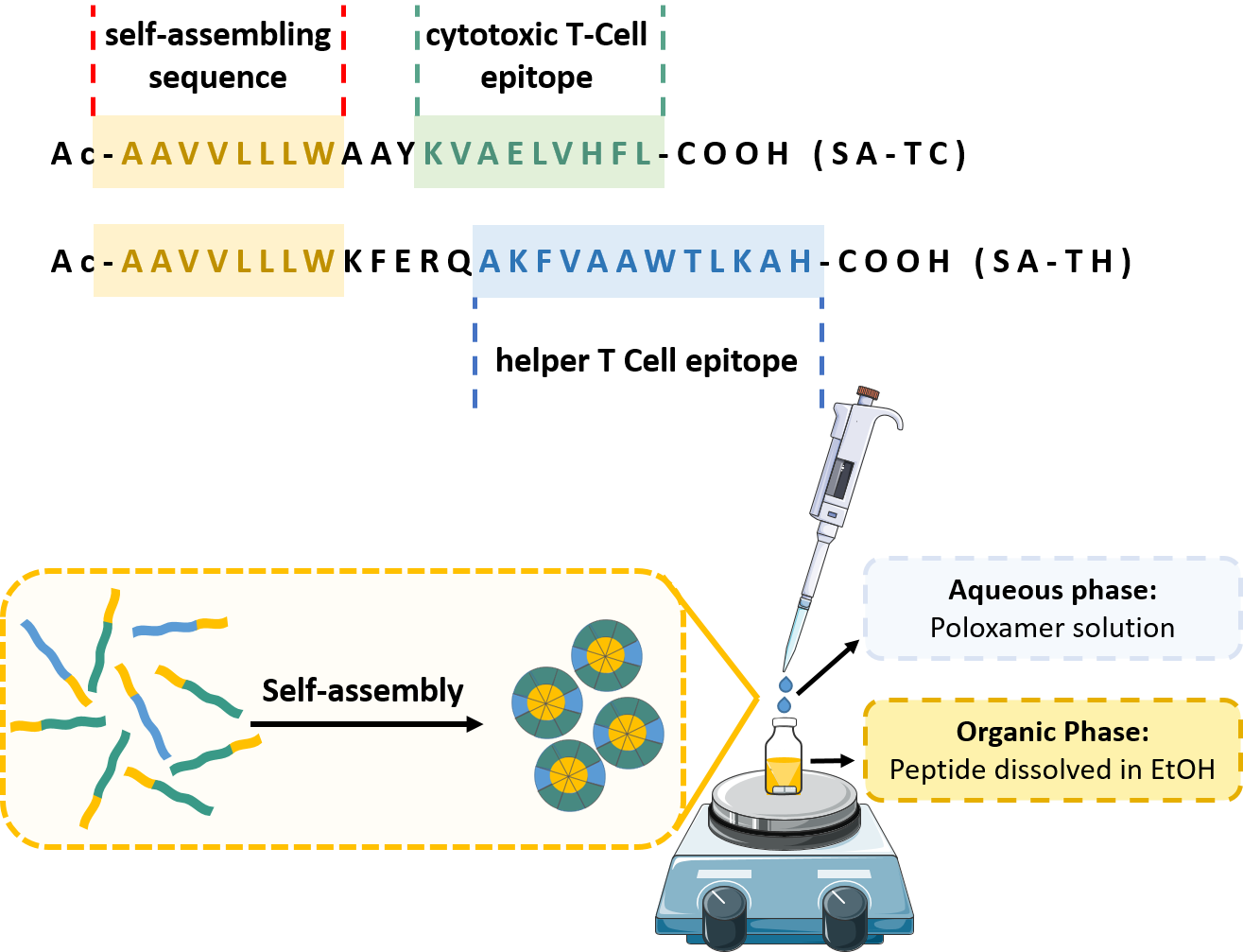


**Fig. S1** Schematic representation of the method to prepare different nanoformulations.

Some parts of the image were reproduced from Servier Medical Art under a Creative Commons Attribution 3.0 Unported License, <https://smart.servier.com/>.


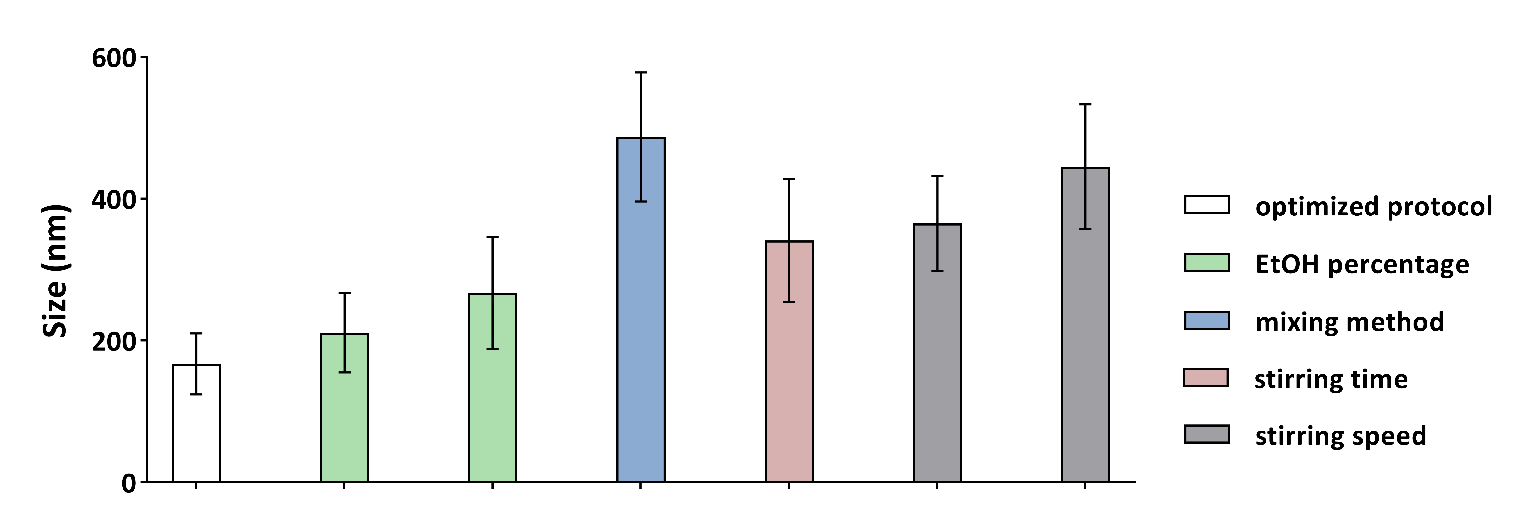


| **EtOH percentage** | 10 | 5 | 20 | -- | -- | -- | -- |
| --- | --- | --- | --- | --- | --- | --- | --- |
| **mixing method** | dropwise | -- | -- | At once | -- | -- | -- |
| **stirring time (min)** | 30 | -- | -- | -- | 15 | -- | -- |
| **stirring speed (rpm)** | 500 | -- | -- | -- | -- | 1000 | 1800 |

**Fig. S2** Effect of different parameters on the particles size. Hyphens stand for the values that are set as in the optimized protocol.


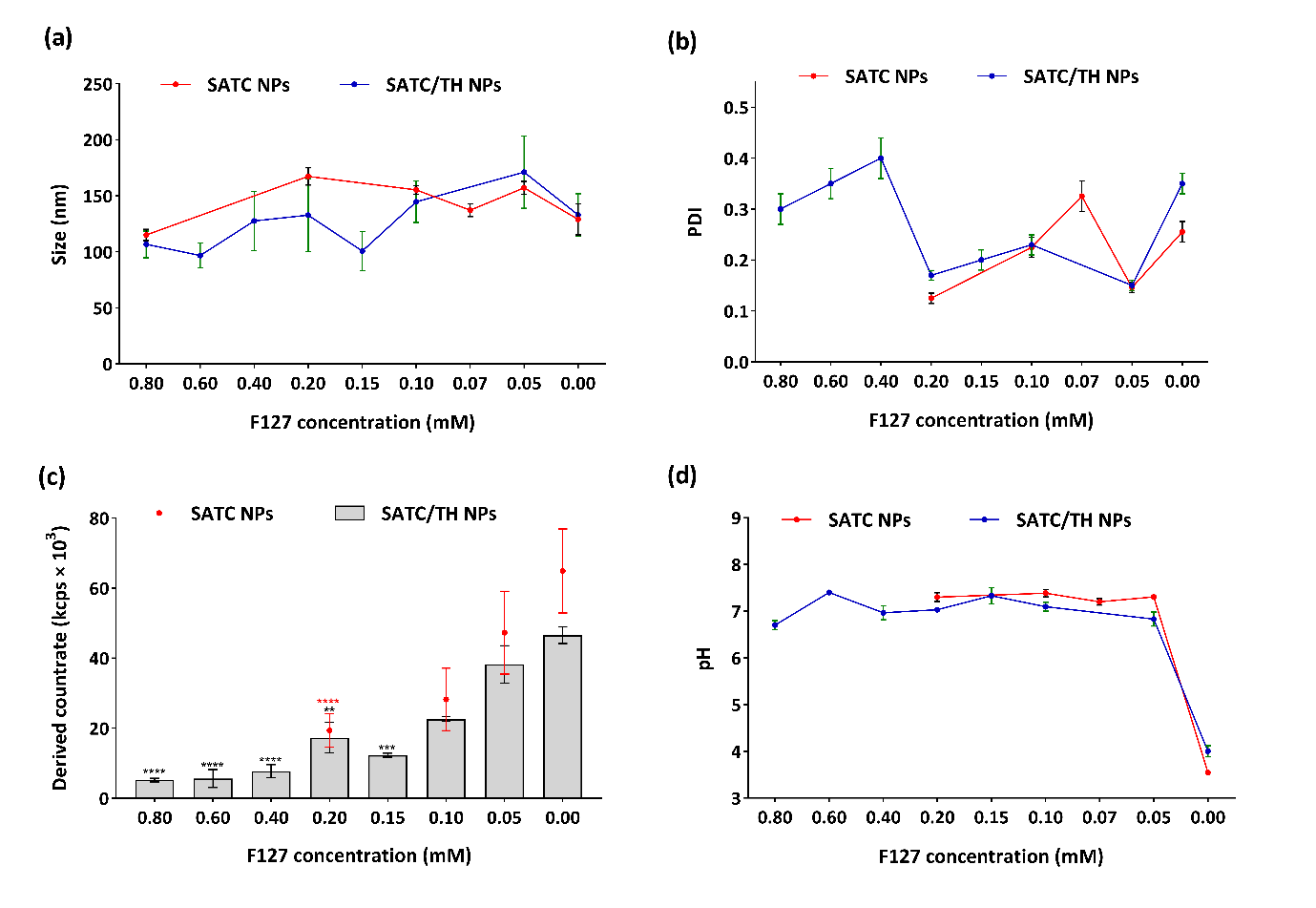


**Fig. S3** (a) size, (b) PDI, (c) derived countrate and (d) pH of the peptide NPs prepared with different F127 concentrations (mean ± S.D., n ≥ 3). Data were analyzed with one-way ANOVA, Tukey test, P < 0.05. The asterisks in (c) express the statistical difference in the countrate of the formulations with 0.05 mM concentration of F127 with the other formulations


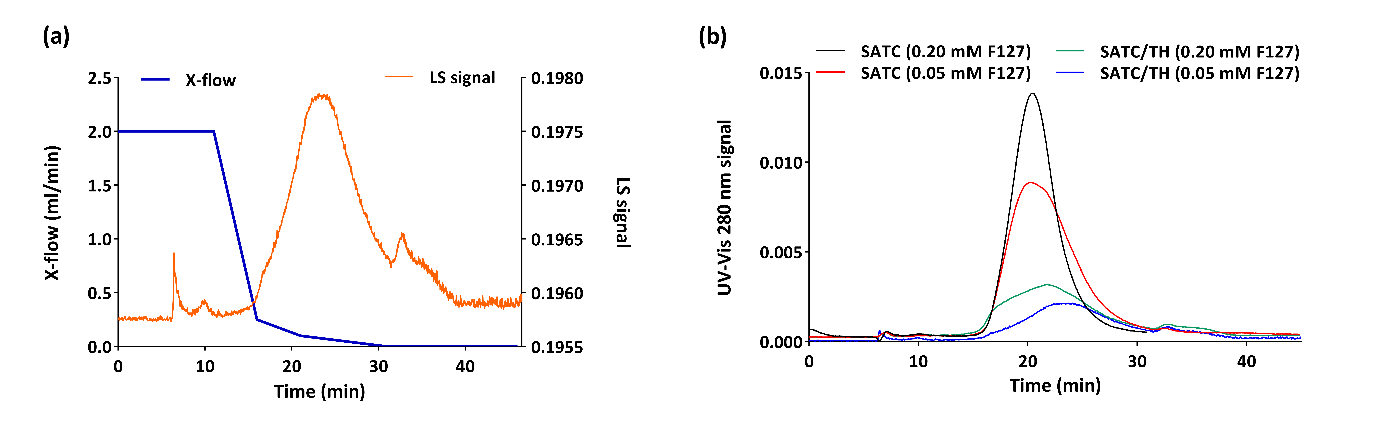


**Fig. S4** (a) AF4 elution program, (b) Fractogram of different NPs recorded by UV detector at 280 nm. The small peak at 5-10 min is the void peak and it is given by non-separated particles, while the peak eluting between 20-27 min corresponds to the nanoparticles.


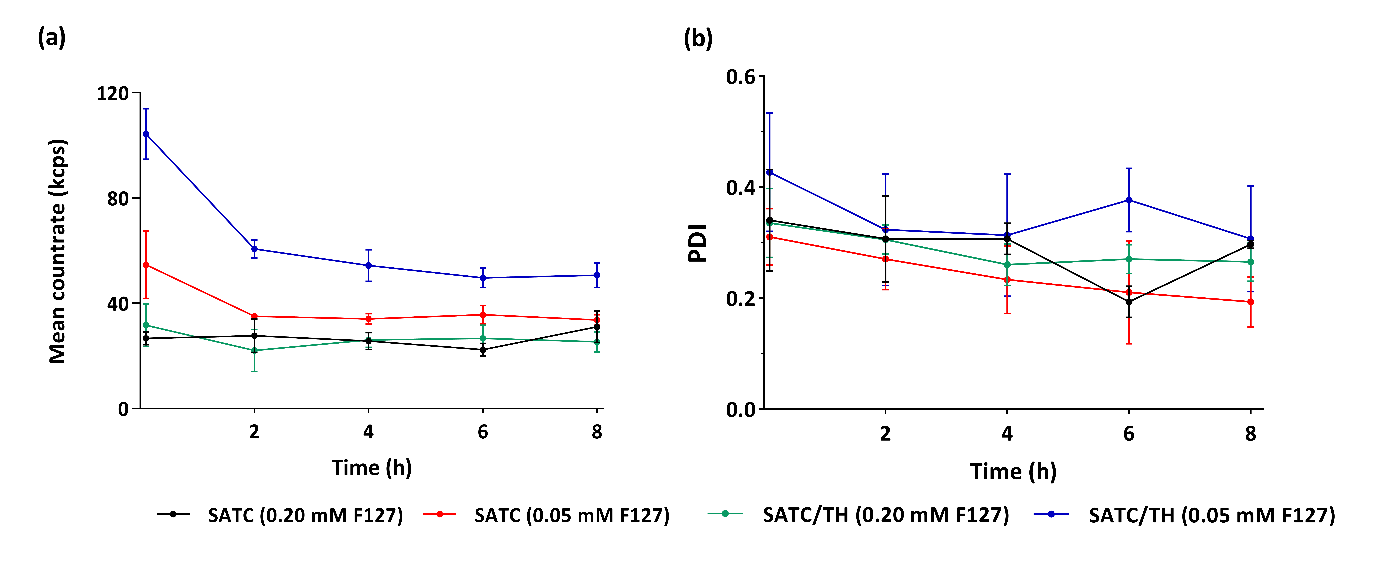


**Fig. S5** (a) Countrate and (b) PDI trends during 8 hours incubation of the NPs in RPMI supplemented with 10% FBS at 37˚C (mean ± S.D., n=3).


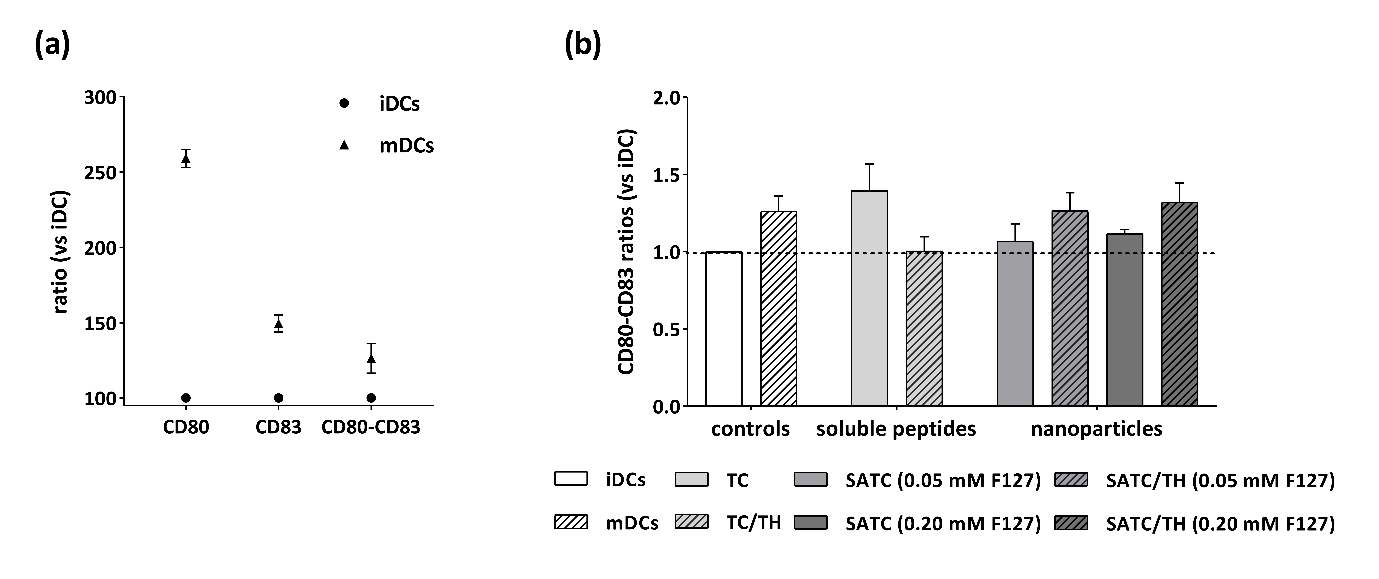


**Fig. S6** (a) Phenotypic changes associated with the maturation of iDCs. (b) Induction of CD80-CD83 double-positive dendritic cell phenotype by incubation of iDCs with NPs at 10 µM for 48 h. Data are shown as the ratio (%) between the mean fluorescence intensity (MFI) of the CD80-CD83 marker in iDCs incubated with the different NPs versus the MFI of iDC incubated in culture media (mean ± S.D., n = 4]). Data were analyzed with one-way ANOVA, Sidak test, P < 0.05.


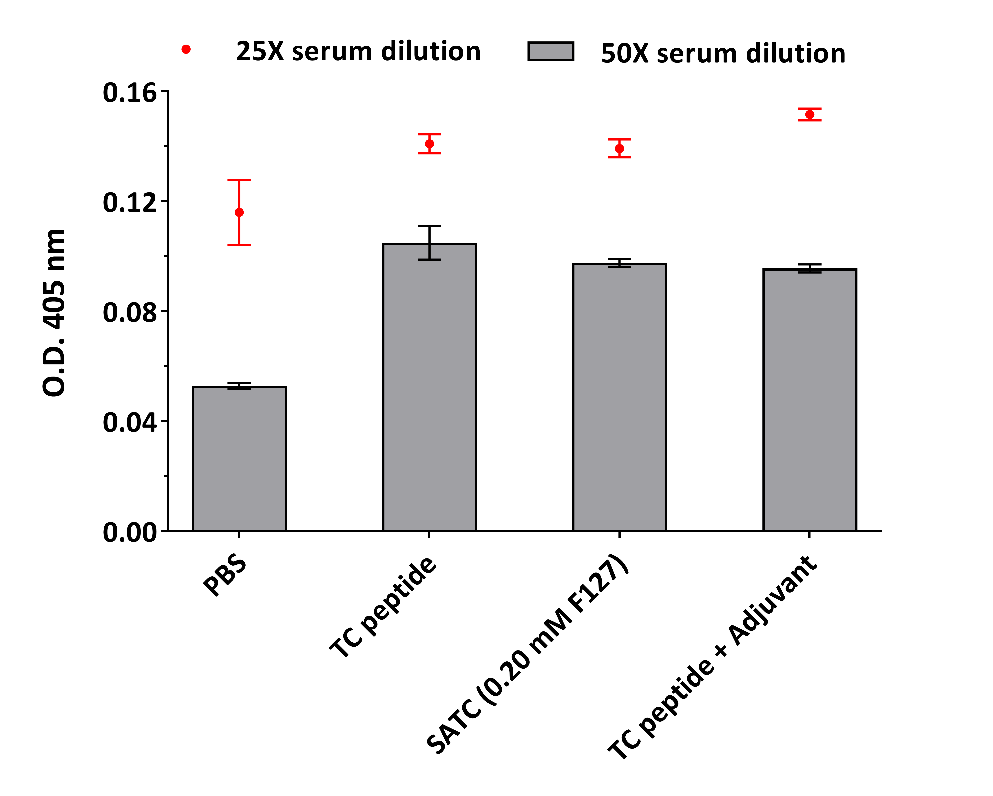


**Fig. S7** Optical densities (λ=405 nm) observed at 25X and 50X mice sera dilutions one week after receiving booster doses at 20000X dilution of the secondary antibody. Data were analyzed with two-way ANOVA, Tukey test, P < 0.05 (mean± S.E., n = 3 mice per group). All groups were statistically different from PBS and equal among them.
